# Molecular characterization of EGFR, PDGFRA and VEGFR2 in cervical adenosquamous carcinoma

**DOI:** 10.1186/1471-2407-9-212

**Published:** 2009-06-29

**Authors:** Adhemar Longatto-Filho, Céline Pinheiro, Olga Martinho, Marise AR Moreira, Luiz FJ Ribeiro, Geraldo S Queiroz, Fernando C Schmitt, Fátima Baltazar, Rui M Reis

**Affiliations:** 1 Life and Health Sciences Research Institute (ICVS), School of Health Sciences, University of Minho, Braga, Portugal; 2 Instituto Adolfo Lutz, São Paulo, SP, Brazil; 3 Department of Pathology of the School of Medicine of the Federal University of Goiás, Goiânia, Go, Brazil; 4 Hospital Araújo Jorge, Goiânia, Go, Brazil; 5 IPATIMUP, Porto, Portugal; 6 School of Medicine of the University of Porto, Porto, Portugal

## Abstract

**Background:**

Adenosquamous carcinoma of the uterine cervix is an infrequent but aggressive subtype of cervical cancer. A better understanding of its biological behaviour is warranted to define more accurate prognosis and therapeutic targets. Currently, the blockage of receptor tyrosine kinase (RTKs) activity is an efficient therapeutic strategy for many different cancers. The objective of this study was to investigate EGFR, PDGFRA and VEGFR2 RTKs overexpression and activating gene mutations in a cohort of 30 adenosquamous carcinomas of the uterine cervix.

**Methods:**

EGFR, PDGFRA and VEGFR2 immunohistochemistry was performed in all samples, followed by DNA isolation from the gross macroscopically dissection of the neoplastic area. Screening for *EGFR *(exons 18–21) and *PDGFRA *(exons 12, 14 and 18) mutations was done by PCR – single-strand conformational polymorphism (PCR-SSCP).

**Results:**

Despite the presence of EGFR immunohistochemical positive reactions in 43% (13/30) of the samples, no *EGFR *activating mutations in the hotspot region (exons 18–21) were identified. A silent base substitution (CAG>CAA) in *EGFR *exon 20 at codon 787 (Q787Q) was found in 17 cases (56%). All PDGFRA immunohistochemical reactions were positive and consistently observed in the stromal component, staining fibroblasts and endothelial cells, as well as in the cytoplasm of malignant cells. No activating *PDGFRA *mutations were found, yet, several silent mutations were observed, such as a base substitution in exon 12 (CCA>CCG) at codon 567 (P567P) in 9 cases and in exon 18 (GTC>GTT) at codon 824 (V824V) in 4 cases. We also observed the presence of base substitutions in intron 14 (IVS14+3G>A and IVS14+49G>A) in two different cases, and in intron 18 (IVS18-50insA) in 4 cases. VEGFR2 positivity was observed in 22 of 30 cases (73.3%), and was significantly associated with lack of metastasis (*p *= 0.038).

**Conclusion:**

This is the most extensive analysis of EGFR, PDGFRA and VEGFR2 in cervical adenosquamous carcinomas. Despite the absence of *EGFR *and *PDGFRA *activating mutations, the presence of overexpression of these three important therapeutic targets in a subset of cases may be important in predicting the sensitivity of adenosquamous carcinoma to specific anti-RTKs drugs.

## Background

Adenosquamous carcinoma (ASC) of the uterine cervix is a relatively infrequent histological subtype of cervical cancer, associated with very aggressive behaviour and reduced survival rates [1]. ASC histopathological interpretation remains controversial; theoretically, ASC is a mixture of malignant glandular and squamous epithelial elements. However, the practical application of this morphological criterion is far from being straightforward and the prognostic significance of the histological alterations is contentious and does not exactly predict the clinical behaviour of ASC [2-4]. The cofactors which contribute to the progression of HPV-infected cervical carcinoma are apparently diverse in each type of histogenetic differentiation. The high risk HPV infection persistence is assumed as a necessary but not sufficient factor to cervical cancer development, and the genetic and molecular disparities involved in the carcinoma progression are still poorly understood [5]. Therefore, a better understanding of ASC biology is needed to identify the key players and potential novel therapeutic strategies.

Disruption of the mitogenic signalling mechanisms, particularly the ones mediated by receptor tyrosine kinases (RTKs), is a hallmark of the carcinogenic process and currently constitutes an important therapeutic target group [6]. RTKs are transmembrane proteins constituted by an extracellular, a transmembrane, a juxtamembrane, and an intracellular domain where two kinase regions are located [7]. Upon growth factor binding, receptor dimerizes and autophosphorylates its intracellular tyrosine residues that activate several downstream signalling cascades, like MAP kinase, PI3-kinase, and JAK/STAT pathways, affecting cellular gene expression [8]. In the neoplastic development and progression, RTKS are commonly deregulated, and excessive phosphorylation sustains signal transduction pathways in an activated state, leading to tumour growth and progression, proliferation, dedifferentiation, inhibition of apoptosis, metastasis and angiogenesis [9,10]. Among the distinct RTK classes, class I [*e.g*. epidermal growth factor receptor (EGFR)] and class III [*e.g*. platelet-derived growth factor receptor-α (PDGFR-α), KIT, vascular endothelial growth factor receptors 1 (VEGFR1), also known as Flt-1, and VEGFR2 or Flk-1] [11] have been consistently implicated in solid neoplasm tumourigenesis.

EGFR was the first RTK to be directly linked to human cancers [12]. The use of EGFR antagonists, namely monoclonal antibodies directed to the extracellular domain, such as Cetuximab (Erbitux^®^) and small molecule tyrosine kinase inhibitors, such as Gefitinib (Iressa^®^) and Erlotinib (Tarceva^®^), have raised great expectations [13]. Recently, several molecular alterations have been associated with patient's response to these new anti-EGFR drugs, in particular, *EGFR *mutations in hotspot regions of the intracellular kinase domain (exons 18–21) were predictive of a positive response to Gefitinib and Erlotinib in a subset of lung cancer [11]. Positive therapeutic results have also been reported using KIT and PDGFRA inhibitors such as Imatinib mesylate (Gleevec^®^) for gastrointestinal stromal tumours (GISTs) [14]. Likewise anti-EGFR drugs, specific gene activating mutations of *KIT *and *PDGFRA *gene seem to predict patients' response to Imatinib [15]. Besides the use of selective inhibitors, promising therapeutic results are being attained with multi-target inhibitors such as Sunitinib (Sutent^®^), which targets KIT, PDGFR, VEGFR2, Sorafenib (Nexavar^®^) that targets KIT, VEGFR2, PDGFR and intracellular tyrosine kinases, such as BRAF [14] and Pazopanib, which targets also KIT, PDGFR and VEGFR [16]. VEGFR2 is not only a mitogenic factor, but essentially an important angiogenic factor; consequently, blocking its activity potentially enhances therapeutic response [17]. Recently, we reported absence of KIT molecular alterations in ASC and provided evidence for KIT activation through KIT/SCF co-expression in a small proportion of cases [18], however there are no similar studies with other RTK therapeutic targets.

The aim of this study was to investigate the presence of EGFR, PDGFRA and VEGFR2 RTKs aberrations, namely overexpression and activating gene mutations in a cohort of 30 adenosquamous carcinomas of the cervix. Accordingly, we intended to assess the potentiality of these RTKs as therapeutic targets in this aggressive tumour type.

## Methods

### Materials

This retrospective study comprised a series of 30 patients with ASC of the uterine cervix, examined and treated at two Hospitals, retrieved from the files of Araújo Jorge Hospital and from the Pathology Department of the School of Medicine of the Federal University of Goiás, Goiania, in Goias State, Brazil, from 1986 to 2000. All histopathological diagnoses were revised by two of the authors (FCS, MARM) and categorized according to the WHO classification [19]. The age of the patients ranged from 24 to 77 years old (mean 49 and median 44.7 years). Clinico-pathological data was available for 29/30 patients and included age at diagnosis, lymph-node and/or distant metastasis, recurrence and overall survival (Table 1). Of these, 12 patients (41.4%) presented lymph-node and/or distant metastasis and 2 (6.9%) presented disease recurrence. The chosen cut-off for follow-up was 24 months, leaving 20 cases for analysis (mean 38.5 and median 33 months). The present study was approved by the local Ethic Committees.

**Table 1:** Clinico-pathological features of ASC patients.

Case	Age	Disease recurrence	Presence of metastasis*	Follow-up (months)	Life status
**1**	41	No	No	64	Alive
**2**	37	No	Yes	22	Alive
**3**	55	NI	NI	NI	NI
**4**	59	No	Yes	16	Dead
**5**	46	No	No	107	Alive
**6**	27	No	No	28	Alive
**7**	33	Yes	No	36	Alive
**8**	67	No	No	34	Alive
**9**	40	No	No	35	Alive
**10**	54	No	Yes	48	Dead
**11**	57	Yes	No	30	Alive
**12**	61	No	No	9	Alive
**13**	67	No	No	32	Alive
**14**	38	No	No	17	Alive
**15**	71	No	No	5	Alive
**16**	40	No	No	87	Alive
**17**	40	No	No	10	Alive
**18**	58	No	Yes	47	Dead
**19**	47	No	No	22	Alive
**20**	41	No	No	14	Alive
**21**	39	No	Yes	9	Dead
**22**	40	No	Yes	48	Alive
**23**	45	No	No	35	Alive
**24**	53	No	Yes	13	Alive
**25**	29	No	Yes	14	Dead
**26**	36	No	Yes	19	Dead
**27**	24	No	Yes	6	Alive
**28**	48	No	Yes	NI	Dead
**29**	56	No	No	22	Alive
**30**	77	No	Yes	17	Alive

* Lymph-node and/or distant metastasis; NI, no information available.

### EGFR, PDGFRA and VEGFR2 Immunohistochemistry

EGFR immunohistochemistry analysis of the present series was previously assessed [20]. For PDGFRA and VEGFR2 expression, immunohistochemistry procedure was performed according to streptavidin-biotin-peroxidase complex principle, using specific antibodies raised against PDGFRA (dilution 1:175, Clone C-20, Santa Cruz Biotechnology, CA), and VEGFR2 (dilution 1:50, Neomarkers, LabVision Corporation, Fremont, CA), as previously described [21-23]. In brief, deparaffinised and rehydrated sections were pre-treated by microwaving in 10 mM citrate buffer (pH 6.0) three times for 5 minutes at 600W. After incubation of VEGFR2 (overnight at 4°C) and PDGFRA (30 minutes at room temperature) primary antibody, the secondary biotinylated goat anti-polyvalent antibody was applied for 10 minutes, followed by incubation with streptavidin-peroxidase complex. The immune reaction was visualised by DAB as a chromogen (Ultravision Detection System Anti-polyvalent, HRP/DAB; LabVision Corporation, Fremont, CA). Appropriated positive and negative controls were included in each run: for PDGFRA, cutaneous-mucosa transition of the anal region, namely medium calibre vessels with a muscular layer was used as positive controls; an angiosarcoma tissue with immunostaining of the blood vessels was used for VEGFR2. For negative controls, primary antibodies were omitted and also replaced by a universal negative control antibody (CEA, rabbit anti-human, DAKO Corporation, Carpinteria, CA). All sections were counterstained with Gill-2 haematoxylin. The immunohistochemical reactions were evaluated as described previously [21]. Briefly, sections were semi-quantitatively scored as follows: (-), 0% of immunoreactive cells; (+), <5% of immunoreactive cells; (++), 5–50% of immunoreactive cells; and (+++), >50% of immunoreactive cells. Samples with scores (-) and (+) were considered negative, and those with scores (++) and (+++) were considered positive.

### DNA isolation

Serial 10 μm unstained sections of paraffin blocks were cut, and one adjacent hematoxylin and eosin-stained section was taken for identification and selection of the tumour tissue. Selected areas containing at least 85% of tumour were marked and, using a sterile needle (Neolus, 25 G-0.5 mm), gross macroscopically dissection was performed. Tissue was placed into a microfuge tube and DNA isolation was performed using Qiagen's QIAamp^® ^DNA Micro Kit (Qiagen, Hilden, Germany), as previously described [18,24].

### Screening for *EGFR *and *PDGFRA *Mutations

Screening for *EGFR *(exons 18–21) and *PDGFRA *(exons 12, 14 and 18) mutations was done by PCR – single-strand conformational polymorphism (PCR-SSCP), as previously described [21,22,25]. Samples showing a mobility shift different from the normal pattern were directly sequenced (Stabvida, Investigation and Services in Biological Sciences Lda, Oeiras, Portugal), as described [25]. All positive cases were confirmed twice with a new and independent PCR amplification, followed by direct sequencing.

### Statistical analysis

Data were stored and analyzed using the SPSS statistical software (version 16.0, SPSS Inc., Chicago, IL). All comparisons were examined for statistical significance using Pearson's chi-square (χ^2^) test and Fisher's exact test (when n < 5), being threshold for significance *p *values < 0.05. Survival curve was plotted using the method of Kaplan and Meier and data compared using the log-rank test.

## Results

Assessment of EGFR, PDGFRA and VEGFR2 overexpression and activating gene mutations was performed in a cohort of 30 adenosquamous carcinomas of the uterine cervix. The results are summarized in Table 2 and are detailed bellow.

**Table 2:** Molecular alterations of EGFR, PDGFRA and VEGFR2 in ASC patients.

Case	EGFR		PDGFRA		VEGFR2
			
	Mutations	IHC*	Mutations	IHC	IHC
**1**	Q787Q	-	P567P; V824V; IVS18-50insA	+++	++
**2**	Normal	+++	Normal	+++	-
**3**	Q787Q	+++	V824V; IVS18-50insA	+++	+++
**4**	Q787Q	+++	V824V; IVS18-50insA	+++	+++
**5**	Q787Q	+++	V824V; IVS18-50insA	+++	+++
**6**	Normal	+	Normal	+++	+++
**7**	Q787Q	+++	P567P; IVS14+3G>A	+++	++
**8**	Q787Q	-	Normal	+++	+++
**9**	Normal	-	Normal	+++	+++
**10**	Normal	+++	P567P	+++	+++
**11**	Normal	-	P567P	np	+++
**12**	Q787Q	-	Normal	+++	+++
**13**	Q787Q	-	Normal	++	+++
**14**	Normal	++	Normal	+++	++
**15**	Q787Q	+	Normal	+++	+++
**16**	Normal	+++	P567P	+++	+
**17**	Q787Q	-	P567P	++	+++
**18**	Normal	-	Normal	+++	+
**19**	Q787Q	+++	Normal	+++	++
**20**	Q787Q	-	Normal	+++	+++
**21**	Normal	+	P567P	np	+++
**22**	Normal	++	P567P	+++	+
**23**	Normal	+++	Normal	++	++
**24**	Q787Q	-	Normal	++	+
**25**	Q787Q	+	IVS14+49G>A	+++	+
**26**	Q787Q	-	P567P	+++	+++
**27**	Normal	-	Normal	np	++
**28**	Q787Q	++	Normal	++	+
**29**	Q787Q	-	Normal	+++	+
**30**	Normal	+++	Normal	++	+++

IHC: Immunohistochemistry; *: previously reported in [20]; np: not possible due to tissue limitation.

### EGFR profile

**Figure 1:**
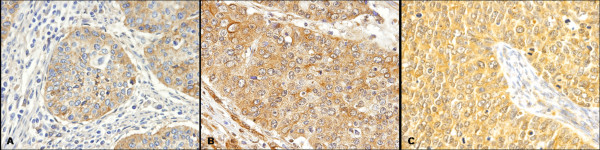
**Immunohistochemical expression of EGFR, PDGFRA and VEGFR2 in cervical adenosquamous carcinoma**. **A) **EGFR positive immunoreaction decorating the cytoplasm of malignant cells. No stromal or endothelial positive reactions were observed (original magnification ×40). **B) **PDGFRA positive immunoreaction was observed in the cytoplasm of malignant cells. PDGFRA positive reaction was also constantly observed in the stromal component, decorating fibroblasts and endothelial cells (original magnification ×40). **C) **VEGFR2 positive immunoreaction was in the cytoplasm of malignant cells. No stromal positive reaction was identified, but positive staining was observed in a few endothelial cells surrounding the neoplastic cells (original magnification ×40).

We have previously found that approximately 43% (13/30) of cases were positive (2+/3+) for EGFR immunohistochemistry (Figure 1A) [20]. In order to determine the molecular basis of such overexpression, we have performed a mutation analysis of cases. No activating mutations in the hotspot region (exons 18–21) of *EGFR *gene were identified. Nevertheless, a silent base substitution (CAG>CAA) in *EGFR *exon 20 at codon 787 (Q787Q) was found in 17 cases (56%) (Table 3).

**Table 3:** Sequence variants of *EGFR *and *PDGFRA *gene in ASC patients.

Gene (exon)	Nucleotide Change	Aminoacid Substitution	N° of cases	dbSNP
***EGFR *(exon 20)**	2361 G>A	Q787Q	17	rs1050171

***PDGFRA *(exon 12)**	1701 G>A	P567P	9	rs1873778

***PDGFRA *(exon 14)**	2002+3G>A	IVS14+3G>A	1	Not yet described
	2002+49G>A	IVS14+49G>A	1	Not yet described

***PDGFRA *(exon 18)**	2472 C>T	V824V	4	rs2228230
	2449-50insA	IVS18-50insA	4	rs3830355

dbSNP: Single Nucleotide Polymorphism database http://www.ncbi.nlm.nih.gov/SNP/

No statistically significant correlations were observed between *EGFR *gene alteration and the clinico-pathological parameters (data not shown).

### PDGFRA profile

PDGFRA immunohistochemical analysis was only possible in 27 out of 30 cases, all of them being positive for PDGFRA expression. Six cases showed moderate positivity (2+) and 21 showed strong positivity (3+) (Figure 1B). Positive immunoreactions were observed in the cytoplasm of malignant cells, and were consistently observed in the stromal component, staining fibroblasts and endothelial cells.

**Figure 2:**
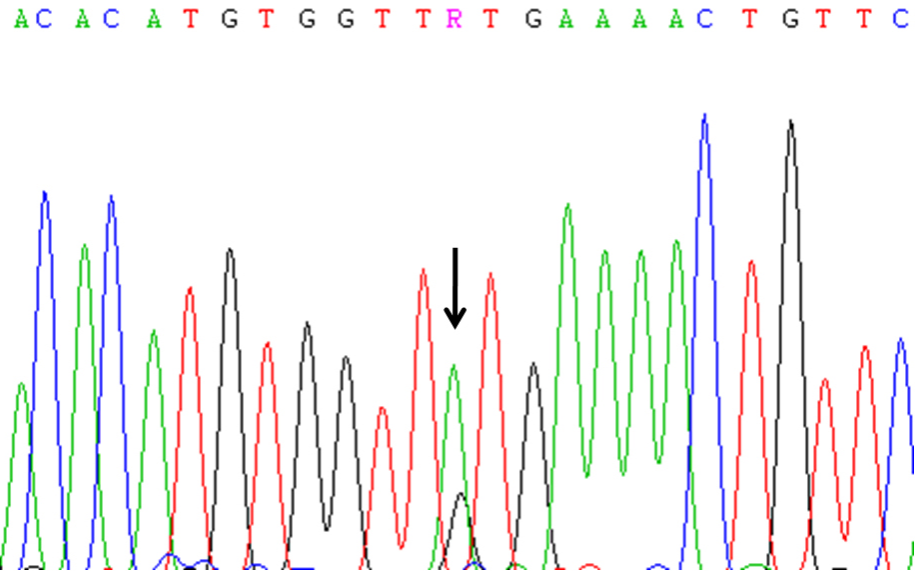
**Electropherogram of part of *PDGFRA *sequence**. DNA sequencing of an intronic base substitution (IVS14+49G>A) in *PDGFRA *intron 14. Arrow indicates G to A transition at 2002 base pairs.

Mutation analysis of *PDGFRA *hotspot exons 12, 14 and 18 of all 30 cases, revealed absence of activating mutations. Yet, we observed several silent mutations, such as a base substitution in exon 12 (CCA>CCG) at codon 567 (P567P) in 9 cases and in exon 18 (GTC>GTT) at codon 824 (V824V) in 4 cases (Table 2). We also observed base substitutions in intron 14 (IVS14+3G>A and IVS14+49G>A) (Figure 2), in two different cases, and in intron 18 (IVS18-50insA) in 4 cases (Table 3).

Since all cases were positive for PDGFRA expression, analysis of correlations with the clinico-pathological data was not performed.

### VEGFR2 profile

VEGFR2 immunohistochemical positivity was observed in 22 of 30 cases (73.3%), with 6 cases showing moderate expression (2+) and 16 cases presenting strong positive immunoreaction (3+). Positive immunoreactions were exclusively observed in the cytoplasm of malignant cells. No stromal positive reaction was identified, but faint positive staining was observed in a few endothelial cells surrounding the neoplastic cells (Figure 1C).

Correlation analysis between VEGFR2 expression and clinico-pathological data revealed a significant association between VEGFR2 overexpression and lack of metastasis (*p *= 0.038). No associations between VEGFR2 expression and overall survival and disease recurrence were found (data not shown).

## Discussion

Advances in the knowledge of the altered molecular events in neoplastic cells have paved the way to the discovery of new and promising targets and drugs for cancer treatment. Three of these potential targets are EGFR, PDGFR and VEGFR2, which have important roles in tumour proliferation and angiogenesis. The results herein reported aimed to identify molecular alterations in these therapeutic targets predictive of a positive response to selective inhibitors in cervical adenosquamous carcinoma (ASC).

Overexpression of EGFR has been reported to be frequent in cervical cancer, ranging from approximately 25–70% [26-31]. Most studies have focused the analysis squamous cell carcinomas [26-31]. We and others have shown that in adenosquamous carcinoma EGFR overexpression varies from 33–43% of cases, being in the range of overall cervical cancer [20,28,29]. Despite the presence of EGFR overexpression, we showed that none of adenosquamous carcinomas harbour *EGFR *gene activating mutations. Nevertheless, a silent base substitution (CAG>CAA) in EGFR exon 20 at codon 787 (Q787Q) was found in 17 cases (56%). This polymorphism is a known single nucleotide polymorphism (SNP), which frequencies vary in different populations, being the G allele more frequent in Asians and African Americans, whereas the A allele is more frequent in Europeans (rs1050171, NCBI SNP database). The implication of this SNP in EGFR function is still unclear. Taguchi *et al*, analysing head and neck squamous cell carcinomas did not observe any significant differences at EGFR mRNA and protein levels in cell lines harbouring different genotypes, despite the described higher sensitivity of (G/A) heterozygous when compared with (G/G) homozygous cell lines to Gefitinib [32]. Recently, Arias-Pulido *et al*, also described the absence of *EGFR *activating mutations in a large series of 89 cervical cancers, including 75 squamous cell carcinomas and 5 adenocarcinomas [33]. The mechanism driving EGFR overexpression in adenosquamous carcinomas remains to be determined. Previous studies have shown that EGFR could be regulated by *EGFR *gene amplification [28] or by HPV oncoproteins, namely the HPV E5 and E6, which are linked with increased EGFR levels, through inhibition of EGFR internalization and degradation [34,35].

Several clinical trials are evaluating the efficacy of anti-EGFR therapies for advanced cervical cancer [6]. Studies of cetuximab-based therapy, either in monotherapy or in association with radiotherapy are ongoing for treatment of recurrent and early cervical carcinoma [Gynecologic Oncology Group (GOG)-0227E; GOG-9918]. A multicenter phase II trial evaluated the clinical outcomes of Gefitinib-based therapy in 30 patients with recurring loco regionally advanced or metastatic cervical cancer [36]. There were no objective responses, however, 1/5 of patients exhibited stable disease, and tumour response was not correlated with EGFR immunohistochemistry levels [36]. These results are not surprising, since it is known that in cancer patients, particularly those with lung cancer, it is the presence of *EGFR *tyrosine kinase activating mutations rather than EGFR immunoreactivity that is associated with a marked clinical response [37]. Since our results indicate the absence of *EGFR *activating mutations in ASC, we would predict that Gefitinib, as well as Erlotinib, in monotherapy are unlikely to be effective in these patients.

Very few studies addressed the role of PDGFRA in cervical carcinogenesis [38-40]. Taja-Chayeb *et al*, have analyzed a total of 36 cases, which included 4 adenosquamous carcinomas. The authors reported overexpression of PDGFRA in neoplastic cells in approximately 42% cases, and a less frequent overexpression in stromal cells (~8%) [38]. In a recent and elegant study, Pietras K *et al*, showed that PDGFRA is almost ubiquitously expressed in the stroma of cervical cancers, but is much less expressed in neoplastic cells [40]. In the present study, we detected PDGFRA overexpression in all cases, either in the neoplastic or stromal component of tumours. These discrepancies could be due in part to the different antibodies used and to distinct histological subtypes analysed. In the present study, no activating mutations were observed, regardless of the presence of several genetic variants, many of them being known as genetic polymorphisms. Our results are in agreement with the described absence of activating mutations in 17 cervical carcinomas [38]. Nevertheless, recent preclinical studies in mouse model and human cervical carcinomas tumour samples, showed significant therapeutic benefits of Imatinib-based therapy [40,41].

VEGFR2 is widely distributed in human tissues and tumours [42]. These receptors were originally thought to be only present in activated endothelial cells; however, recent immunohistochemical studies showed that VEGFR2 is also present in cancer cells and that translocation of phosphorylated VEGFR2 to the nuclei is a frequent event presumably being linked to an existing autocrine VEGF/VEGFR2 loop [42-44]. An interesting recent report suggests that VEGFR2 is a marker of precancerous stem cells [45]. To the best of our knowledge, there are no reports on VEGFR2 expression in cervical adenosquamous carcinomas. We showed presence of neoplastic VEGFR2 expression in approximately 2/3 of cases. Our data showed an association between VEGFR2 overexpression and lack of metastasis. This apparently paradox may suggest that other alternative molecules can drive the metastatic spread in this rare type of cervical cancer, even when VEGFR2 is overexpressed in the cancer cell cytoplasm, as observed in the present study. Several clinical trials of anti-VEGFR2 drugs are being conducted in cervical cancer [6]. Sorafenib, is being assessed in a phase I/II clinical trial in combination with radiotherapy and cisplatin (DDPDRO-002) [46]. Presently, simultaneous inhibition of several receptors tyrosine kinases is believed to optimize the overall therapeutic benefit associated with molecular targeted anticancer agents [47]. A clinical study is ongoing to evaluate the efficacy and safety of Pazopanib and Lapatinib, a dual tyrosine kinase inhibitor of EGFR and HER2, alone or in combination in patients with metastatic cervical cancer (VEG105281) [48].

## Conclusion

In conclusion, the present study is most the comprehensive analysis of EGFR, PDGFRA and VEGFR2 oncogenes in adenosquamous carcinoma. We observed absence of activating mutations in *EGFR *and *PDGFRA *oncogenes, despite the presence of protein overexpression. VEGR2 was frequently overexpressed and associated with lack of metastasis. The current molecular profiling can be valuable for future selection of adenosquamous cervical carcinoma therapeutic options.

## Authors' contributions

ALF and RMR were responsible for study concept and design, study supervision, and manuscript drafting and critical revision. CP, OM and FB performed the molecular genetic studies, immunohistochemistry reactions and participated in the drafting of the manuscript. MARM, LFJR and GSQ were responsible for clinico-pathological collection and drafting of the manuscript. FCS and ALF assessed immunohistochemistry results and drafting of the manuscript. All the authors read and approved the final manuscript.

## Pre-publication history

The pre-publication history for this paper can be accessed here:

http://www.biomedcentral.com/1471-2407/9/212/prepub
